# 1-Ammonio­naphthalene-2-sulfonate

**DOI:** 10.1107/S1600536809036538

**Published:** 2009-09-19

**Authors:** Ayyaz Mahmood, Ayoub Rashid, Muhammad Nadeem Arshad, Hafiz Muhammad Adeel Sharif, Islam Ullah Khan

**Affiliations:** a Materials Chemistry Laboratory, Department of Chemistry, GC University, Lahore 54000, Pakistan

## Abstract

In the mol­ecule of the zwitterionic title compound, C_10_H_9_NO_3_S, an intra­molecular N—H⋯O hydrogen bond results in the formation of an almost planar six-membered ring (r.m.s daviation = 0.0150 Å), which is oriented at a dihedral angle of 1.63 (3)° with respect to the naphthalene ring system. In the crystal structure, inter­molecular N—H⋯O hydrogen bonds link the mol­ecules into a two-dimensional network.

## Related literature

For general background to the use of amino naphthalene sulfonic acid derivatives as a inter­mediates for the syntheses of azo dyes, see: O’Neil (2001 ▶). For related structures, see: Arshad *et al.* (2008*a* ▶,*b* ▶); Genther *et al.* (2007 ▶); Shafiq *et al.* (2008 ▶); Smith *et al.* (2004 ▶, 2009 ▶). For bond-length data, see: Allen *et al.* (1987 ▶). 
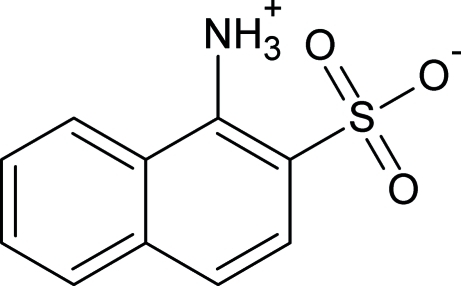


## Experimental

### 

#### Crystal data

C_10_H_9_NO_3_S
*M* *_r_* = 223.24
Orthorhombic, 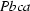

*a* = 9.4337 (3) Å
*b* = 10.6359 (4) Å
*c* = 18.6775 (6) Å
*V* = 1874.02 (11) Å^3^
*Z* = 8
Mo *K*α radiation
μ = 0.33 mm^−1^
*T* = 296 K
0.29 × 0.21 × 0.18 mm

#### Data collection

Bruker Kappa APEXII CCD area-detector diffractometer
Absorption correction: multi-scan (*SADABS*; Bruker, 2007 ▶) *T*_min_ = 0.911, *T*_max_ = 0.943
10742 measured reflections
2326 independent reflections
1763 reflections with *I* > 2/s(*I*)
*R*_int_ = 0.035

#### Refinement

*R*[*F* ^2^ > 2σ(*F* ^2^)] = 0.038
*wR*(*F* ^2^) = 0.107
*S* = 1.03
2326 reflections
145 parameters
H atoms treated by a mixture of independent and constrained refinement
Δρ_max_ = 0.30 e Å^−3^
Δρ_min_ = −0.50 e Å^−3^

### 

Data collection: *APEX2* (Bruker, 2007 ▶); cell refinement: *SAINT* (Bruker, 2007 ▶); data reduction: *SAINT*; program(s) used to solve structure: *SHELXS97* (Sheldrick, 2008 ▶); program(s) used to refine structure: *SHELXL97* (Sheldrick, 2008 ▶); molecular graphics: *ORTEP-3 for Windows* (Farrugia, 1997 ▶) and *PLATON* (Spek, 2009 ▶); software used to prepare material for publication: *WinGX* (Farrugia, 1999 ▶) and *PLATON*.

## Supplementary Material

Crystal structure: contains datablocks I, New_Global_Publ_Block. DOI: 10.1107/S1600536809036538/hk2764sup1.cif

Structure factors: contains datablocks I. DOI: 10.1107/S1600536809036538/hk2764Isup2.hkl

Additional supplementary materials:  crystallographic information; 3D view; checkCIF report

## Figures and Tables

**Table 1:** Hydrogen-bond geometry (Å, °)

*D*—H⋯*A*	*D*—H	H⋯*A*	*D*⋯*A*	*D*—H⋯*A*
N1—H1*N*⋯O1^i^	0.86 (2)	1.94 (2)	2.762 (2)	160.4 (18)
N1—H2*N*⋯O2	0.91 (2)	1.83 (2)	2.651 (2)	149.0 (19)
N1—H3*N*⋯O3^ii^	0.87 (3)	2.14 (3)	2.982 (2)	162 (2)

Symmetry codes: (i) 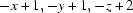; (ii) 
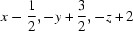
.

**Fig. 1.**
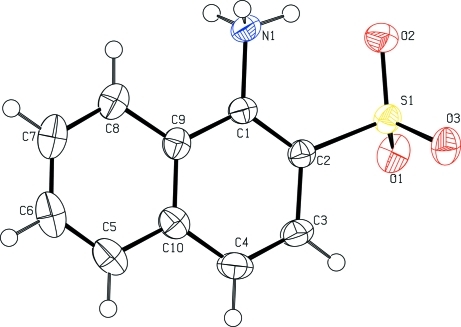
The molecular structure of the title molecule, with the atom-numbering scheme. Displacement ellipsoids are drawn at the 50% probability level.

**Fig. 2.**
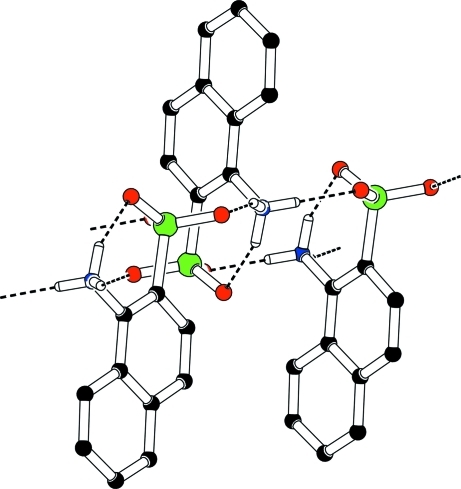
A partial packing diagram. Hydrogen bonds are shown as dashed lines. Hydrogen atoms not involved in hydrogen bonding have been omitted for clarity.

## supplementary crystallographic information

### Comment

The title compound is a zwitterion of 1-amino-2-naphthalene sulfonic acid (*o*-naphthionic acid). Amino naphthalene sulfonic acid derivatives have been used as an intermediate for the syntheses of azo dyes (O'Neil, 2001) and gained importance in complexation (Genther *et al.*, 2007). We purchased 1-amino-2-naphthalene sulfonic acid to use as a precursor for the syntheses of biologically active thiazine related heterocycles (Arshad *et al.*, 2008*a*, *b*; Shafiq *et al.*, 2008). The crystal structures of 5-aminonaphthalene-1-sulfonic acid (Genther *et al.*, 2007; Smith *et al.*, 2004) and 8-ammonionaphthalene-2-sulfonate monohydrate (Smith *et al.*, 2009) have already been published, which are position isomers of the title compound.

In the molecule of the title compound, (Fig. 1), the bond lengths (Allen *et al.*, 1987) and angles are within normal ranges. Rings A (C1-C4/C9/C10) and B (C5-C10) are, of course, planar. The dihedral angles between them are A/B = 1.94 (3)°. The intramolecular N-H···O hydrogen bond (Table 1) results in the formation of a planar six-membered ring C (S1/O2/N1/C1/C2/H2N), which is oriented with respect to the other rings at dihedral angles of A/C = 0.74 (3) and B/C = 2.59 (3) °. So, the rings are almost coplanar.

In the crystal structure, intermolecular N-H···O hydrogen bonds (Table 1) link the molecules into a two-dimensional network (Fig. 2), in which they may be effective in the stabilization of the structure.

### Experimental

The title compound was purchased from Sigma-Aldrich and recrystalized in methanol for X-ray analysis.

### Refinement

Atoms H1N, H2N and H3N (for NH_3_) are located in a difference Fourier map and constrained to ride on their parent atom, with U_iso_(H) = 1.2U_eq_(N). The remaining H atoms were positioned geometrically with C-H = 0.93 Å for aromatic H atoms and constrained to ride on their parent atoms, with U_iso_(H) = 1.2U_eq_(C).

### Crystal data

**Table:**

C_10_H_9_NO_3_S	*F*(000) = 928
*M**_r_* = 223.24	*D*_x_ = 1.582 Mg m^−^^3^
Orthorhombic, *P**b**c**a*	Mo *K*α radiation, λ = 0.71073 Å
Hall symbol: -P 2ac 2ab	Cell parameters from 2779 reflections
*a* = 9.4337 (3) Å	θ = 3.1–27.5°
*b* = 10.6359 (4) Å	µ = 0.33 mm^−^^1^
*c* = 18.6775 (6) Å	*T* = 296 K
*V* = 1874.02 (11) Å^3^	Hexagonal, dark brown
*Z* = 8	0.29 × 0.21 × 0.18 mm

### Data collection

**Table:**

Bruker Kappa APEXII CCD area-detector diffractometer	2326 independent reflections
Radiation source: fine-focus sealed tube	1763 reflections with *I* > 2/s(*I*)
graphite	*R*_int_ = 0.035
φ and ω scans	θ_max_ = 28.3°, θ_min_ = 3.1°
Absorption correction: multi-scan (*SADABS*; Bruker, 2007)	*h* = −12→12
*T*_min_ = 0.911, *T*_max_ = 0.943	*k* = −14→8
10742 measured reflections	*l* = −24→24

### Refinement

**Table:**

Refinement on *F*^2^	Primary atom site location: structure-invariant direct methods
Least-squares matrix: full	Secondary atom site location: difference Fourier map
*R*[*F*^2^ > 2σ(*F*^2^)] = 0.038	Hydrogen site location: inferred from neighbouring sites
*wR*(*F*^2^) = 0.107	H atoms treated by a mixture of independent and constrained refinement
*S* = 1.03	*w* = 1/[σ^2^(*F*_o_^2^) + (0.0546*P*)^2^ + 0.6846*P*] where *P* = (*F*_o_^2^ + 2*F*_c_^2^)/3
2326 reflections	(Δ/σ)_max_ = 0.001
145 parameters	Δρ_max_ = 0.30 e Å^−^^3^
0 restraints	Δρ_min_ = −0.50 e Å^−^^3^

### Special details

**Table:**

Geometry. All e.s.d.'s (except the e.s.d. in the dihedral angle between two l.s. planes) are estimated using the full covariance matrix. The cell e.s.d.'s are taken into account individually in the estimation of e.s.d.'s in distances, angles and torsion angles; correlations between e.s.d.'s in cell parameters are only used when they are defined by crystal symmetry. An approximate (isotropic) treatment of cell e.s.d.'s is used for estimating e.s.d.'s involving l.s. planes.
Refinement. Refinement of *F*^2^ against ALL reflections. The weighted *R*-factor *wR* and goodness of fit *S* are based on *F*^2^, conventional *R*-factors *R* are based on *F*, with *F* set to zero for negative *F*^2^. The threshold expression of *F*^2^ > σ(*F*^2^) is used only for calculating *R*-factors(gt) *etc*. and is not relevant to the choice of reflections for refinement. *R*-factors based on *F*^2^ are statistically about twice as large as those based on *F*, and *R*- factors based on ALL data will be even larger.

### Fractional atomic coordinates and isotropic or equivalent isotropic displacement parameters (Å^2^)

**Table:**

	*x*	*y*	*z*	*U*_iso_*/*U*_eq_	
S1	0.48056 (5)	0.72906 (4)	1.02833 (2)	0.02659 (15)	
O1	0.62861 (15)	0.69891 (13)	1.04059 (8)	0.0375 (4)	
O2	0.38420 (16)	0.65642 (14)	1.07238 (7)	0.0401 (4)	
O3	0.45788 (15)	0.86350 (13)	1.03188 (7)	0.0369 (3)	
N1	0.25407 (18)	0.53879 (16)	0.96588 (9)	0.0282 (3)	
H1N	0.270 (2)	0.459 (2)	0.9642 (10)	0.034*	
H2N	0.273 (2)	0.565 (2)	1.0112 (12)	0.034*	
H3N	0.166 (3)	0.558 (2)	0.9579 (11)	0.034*	
C1	0.34654 (18)	0.60250 (16)	0.91466 (9)	0.0248 (4)	
C2	0.44755 (18)	0.68602 (17)	0.93750 (9)	0.0253 (4)	
C3	0.5354 (2)	0.7463 (2)	0.88661 (11)	0.0346 (4)	
H3	0.6054	0.8017	0.9018	0.041*	
C4	0.5178 (2)	0.7236 (2)	0.81550 (11)	0.0373 (5)	
H4	0.5756	0.7646	0.7826	0.045*	
C5	0.3947 (2)	0.6159 (2)	0.71689 (11)	0.0411 (5)	
H5	0.4495	0.6590	0.6836	0.049*	
C6	0.2971 (3)	0.5317 (2)	0.69409 (12)	0.0455 (6)	
H6	0.2852	0.5175	0.6453	0.055*	
C7	0.2144 (3)	0.4659 (2)	0.74345 (12)	0.0457 (5)	
H7	0.1486	0.4074	0.7272	0.055*	
C8	0.2287 (2)	0.48638 (19)	0.81515 (11)	0.0373 (5)	
H8	0.1727	0.4418	0.8473	0.045*	
C9	0.32805 (19)	0.57488 (17)	0.84084 (10)	0.0277 (4)	
C10	0.4142 (2)	0.63928 (18)	0.79073 (10)	0.0317 (4)	

### Atomic displacement parameters (Å^2^)

**Table:**

	*U*^11^	*U*^22^	*U*^33^	*U*^12^	*U*^13^	*U*^23^
S1	0.0286 (2)	0.0233 (2)	0.0278 (2)	−0.00027 (18)	−0.00188 (17)	0.00027 (17)
O1	0.0327 (7)	0.0331 (7)	0.0467 (8)	0.0037 (6)	−0.0106 (6)	−0.0016 (6)
O2	0.0467 (9)	0.0439 (8)	0.0297 (7)	−0.0125 (7)	0.0029 (6)	0.0025 (6)
O3	0.0451 (8)	0.0254 (7)	0.0402 (8)	0.0056 (6)	−0.0033 (6)	−0.0043 (6)
N1	0.0271 (8)	0.0231 (8)	0.0343 (9)	−0.0033 (7)	0.0033 (7)	0.0014 (7)
C1	0.0224 (8)	0.0216 (8)	0.0303 (9)	0.0027 (7)	0.0034 (7)	0.0015 (7)
C2	0.0252 (8)	0.0231 (8)	0.0277 (9)	0.0012 (7)	0.0008 (7)	0.0000 (7)
C3	0.0301 (9)	0.0379 (11)	0.0357 (10)	−0.0108 (8)	0.0024 (8)	0.0019 (8)
C4	0.0364 (10)	0.0415 (12)	0.0339 (10)	−0.0053 (9)	0.0087 (8)	0.0057 (9)
C5	0.0490 (12)	0.0454 (12)	0.0290 (10)	0.0119 (11)	0.0036 (9)	0.0006 (9)
C6	0.0558 (13)	0.0454 (13)	0.0353 (11)	0.0151 (11)	−0.0103 (10)	−0.0111 (9)
C7	0.0502 (13)	0.0377 (12)	0.0493 (13)	0.0010 (10)	−0.0143 (10)	−0.0119 (10)
C8	0.0360 (11)	0.0323 (10)	0.0435 (11)	−0.0026 (9)	−0.0048 (8)	−0.0044 (9)
C9	0.0259 (8)	0.0244 (9)	0.0328 (10)	0.0051 (7)	−0.0016 (7)	−0.0023 (7)
C10	0.0322 (9)	0.0323 (10)	0.0305 (10)	0.0055 (8)	0.0005 (7)	0.0004 (8)

### Geometric parameters (Å, °)

**Table:**

S1—O3	1.4473 (14)	C4—C10	1.405 (3)
S1—O2	1.4491 (14)	C4—H4	0.9300
S1—O1	1.4513 (14)	C5—C6	1.353 (3)
S1—C2	1.7845 (18)	C5—C10	1.413 (3)
N1—C1	1.461 (2)	C5—H5	0.9300
N1—H1N	0.86 (2)	C6—C7	1.395 (3)
N1—H2N	0.91 (2)	C6—H6	0.9300
N1—H3N	0.86 (2)	C7—C8	1.363 (3)
C1—C2	1.371 (2)	C7—H7	0.9300
C1—C9	1.420 (2)	C8—C9	1.412 (3)
C2—C3	1.415 (3)	C8—H8	0.9300
C3—C4	1.360 (3)	C9—C10	1.416 (3)
C3—H3	0.9300		
			
O3—S1—O2	114.07 (9)	C3—C4—C10	121.32 (18)
O3—S1—O1	110.69 (9)	C3—C4—H4	119.3
O2—S1—O1	113.34 (9)	C10—C4—H4	119.3
O3—S1—C2	105.74 (8)	C6—C5—C10	120.8 (2)
O2—S1—C2	107.07 (8)	C6—C5—H5	119.6
O1—S1—C2	105.15 (8)	C10—C5—H5	119.6
C1—N1—H1N	109.3 (14)	C5—C6—C7	120.3 (2)
C1—N1—H2N	110.2 (14)	C5—C6—H6	119.9
H1N—N1—H2N	107.7 (19)	C7—C6—H6	119.9
C1—N1—H3N	110.5 (14)	C8—C7—C6	120.9 (2)
H1N—N1—H3N	113 (2)	C8—C7—H7	119.5
H2N—N1—H3N	106.3 (19)	C6—C7—H7	119.5
C2—C1—C9	121.43 (16)	C7—C8—C9	120.4 (2)
C2—C1—N1	120.77 (16)	C7—C8—H8	119.8
C9—C1—N1	117.79 (16)	C9—C8—H8	119.8
C1—C2—C3	119.45 (17)	C8—C9—C10	118.59 (17)
C1—C2—S1	125.70 (14)	C8—C9—C1	123.31 (18)
C3—C2—S1	114.85 (14)	C10—C9—C1	118.10 (16)
C4—C3—C2	120.28 (18)	C4—C10—C5	121.61 (19)
C4—C3—H3	119.9	C4—C10—C9	119.36 (17)
C2—C3—H3	119.9	C5—C10—C9	119.03 (19)
			
C9—C1—C2—C3	0.1 (3)	C6—C7—C8—C9	0.0 (3)
N1—C1—C2—C3	−179.95 (17)	C7—C8—C9—C10	−1.4 (3)
C9—C1—C2—S1	179.69 (14)	C7—C8—C9—C1	178.78 (19)
N1—C1—C2—S1	−0.3 (3)	C2—C1—C9—C8	177.77 (18)
O3—S1—C2—C1	−120.22 (16)	N1—C1—C9—C8	−2.2 (3)
O2—S1—C2—C1	1.78 (19)	C2—C1—C9—C10	−2.0 (3)
O1—S1—C2—C1	122.61 (16)	N1—C1—C9—C10	178.00 (16)
O3—S1—C2—C3	59.42 (16)	C3—C4—C10—C5	179.5 (2)
O2—S1—C2—C3	−178.58 (14)	C3—C4—C10—C9	−1.2 (3)
O1—S1—C2—C3	−57.75 (16)	C6—C5—C10—C4	178.0 (2)
C1—C2—C3—C4	1.4 (3)	C6—C5—C10—C9	−1.2 (3)
S1—C2—C3—C4	−178.30 (17)	C8—C9—C10—C4	−177.23 (18)
C2—C3—C4—C10	−0.8 (3)	C1—C9—C10—C4	2.6 (3)
C10—C5—C6—C7	−0.3 (3)	C8—C9—C10—C5	2.0 (3)
C5—C6—C7—C8	0.9 (3)	C1—C9—C10—C5	−178.18 (17)

### Hydrogen-bond geometry (Å, °)

**Table:**

*D*—H···*A*	*D*—H	H···*A*	*D*···*A*	*D*—H···*A*
N1—H1N···O1^i^	0.86 (2)	1.94 (2)	2.762 (2)	160.4 (18)
N1—H2N···O2	0.91 (2)	1.83 (2)	2.651 (2)	149.0 (19)
N1—H3N···O3^ii^	0.87 (3)	2.14 (3)	2.982 (2)	162 (2)

Symmetry codes: (i) −*x*+1, −*y*+1, −*z*+2; (ii) *x*−1/2, −*y*+3/2, −*z*+2.
